# Changes in horizontal and vertical peripheral refraction and their predictive value for myopia control after orthokeratology: a 2-year prospective study

**DOI:** 10.3389/fmed.2026.1893370

**Published:** 2026-08-18

**Authors:** Renai Chen, Aiqin Xu, Shuyu Zhang, Jian Wang, Yuting Zhang, Lihua Yu, Yan Lian, Wanqing Jin

**Affiliations:** 1 National Clinical Research Center for Ocular Diseases, Eye Hospital, Wenzhou Medical University, Wenzhou, China; 2 School of Ophthalmology & Optometry, Wenzhou Medical University, Wenzhou, Zhejiang, China

**Keywords:** axial elongation, myopia, orthokeratology, peripheral astigmatism component, peripheral refraction

## Abstract

**Purpose:**

To investigate changes in peripheral refraction in both horizontal and vertical meridians after long-term orthokeratology and their correlation with axial elongation (AE).

**Methods:**

Children aged 8 to 14 years were fitted with orthokeratology lenses. Axial length and peripheral refraction, from 12 locations in the horizontal and vertical meridians, were measured at baseline and every 6 months over the study period.

**Results:**

39 subjects were recruited, with a mean AE of 0.38 ± 0.17 mm at 24 months. Except superior 5° (S5°), relative peripheral refraction (RPR) showed significant myopic defocus at all locations (all *p* ≤ 0.003) after orthokeratology. Except temporal 20° (T20°) and 30° (T30°), peripheral vertical (90°)/horizontal (180°) astigmatism component (J_0_) changed significantly (all *p* ≤ 0.045) and except nasal 30° (N30°), peripheral oblique (45° and 135°) astigmatism component (J_45_) changed significantly (all *p* ≤ 0.022). Except 18 months, the AE of each follow-up was negatively correlated with J_0_ at inferior 5° (I5°) (*r* = −0.371, −0.364, −0.496, *p* = 0.033, 0.041, 0.007, respectively), J_45_ at N10° (*r* = −0.401, −0.348, −0.417, *p* = 0.019, 0.048, 0.024, respectively) and I10° (*r* = −0.415, −0.512, −0.471, *p* = 0.018, 0.003, 0.011, respectively), as measured at 6 months. The 18-month AE was only negatively correlated with the 6-month J_45_ at I10° (*r* = −0.415, *p* = 0.031). Multiple linear regression models were established to assess the relationship between AE and the 6-month J_0_ at I5°, J_45_ at N10°, J_45_ at I10° (*r*^2^ = 0.340, 0.336, 0.263, 0.430, *p* = 0.010, 0.013, 0.077, 0.004, respectively).

**Conclusion:**

Significant changes in RPR, J_0_ and J_45_ were observed at most locations in the horizontal and vertical meridians after orthokeratology. Partial peripheral astigmatism components measured at 6 months demonstrated significant predictive value for the long-term efficacy of orthokeratology in myopia control.

## Introduction

In the last decade, the global incidence of myopia has increased significantly, particularly in East and Southeast Asia (1, 2). It was expected that by 2050, the proportion of population with myopia and high myopia may reach 49.8% and 9.8% worldwide, respectively (3). A series of complications associated with high myopia were the sixth leading cause of blindness (4). Therefore, the prevention and control of myopia have attracted increasing attention and become a research priority in ophthalmology.

Orthokeratology is one of the mainstream methods for myopia control, with numerous studies demonstrating its efficacy in slowing axial elongation (AE) in children (5–7). However, the mechanism by which orthokeratology lenses (OKL) retard myopia progression remains unclear. One widely accepted hypothesis is that the myopic defocus signals of the peripheral retina induced by OKL may be a key component in reducing axial growth. It is believed that the reverse-geometry design of OKL reshapes the cornea, resulting in central epithelial thinning and mid-peripheral thickening (8–11). These morphological changes correct central refractive error and induce myopic defocus in the peripheral retina (12, 13).

It is still controversial whether there is a correlation between AE and peripheral refraction after orthokeratology. Jakobsen et al. (14) investigated relative peripheral refraction (RPR) and axial length (AL) in the horizontal meridian before and after orthokeratology. They found that OKL caused significant myopic defocus in the peripheral retina, but found no correlation between AE and the changes in 6-month RPR during the 18-month follow-up period. Gifford et al. (15) also did not find any correlation between AE and the changes in horizontal RPR after 12-month orthokeratology. However, Chen et al. (16) reported significant correlations between AE and the changes in RPR at both nasal 30° (N30°) and temporal 30° (T30°) retinal locations after 3 months of OKL wear. Wang et al. (17) also found a correlation between AE and the change in RPR at N20° after 24-month orthokeratology. Therefore, the correlation between AE and RPR is inconsistent across studies with different wearing times, which poses a challenge to the central premise that orthokeratology inhibits myopia progression primarily via peripheral retinal myopic defocus. Meanwhile, most previous studies on peripheral refraction after orthokeratology focused on the horizontal meridian. Only a few studies reported the vertical meridian and their subjects were adults with short-term wear of OKL (13, 18, 19). Therefore, the purpose of this study was to investigate the changes in peripheral refraction in both horizontal and vertical meridians and their correlation with AE in myopic children undergoing long-term orthokeratology.

## Methods

### Study design

The protocol of the study followed the tenets of the Declaration of Helsinki, and was approved by the Ethics Committee of the Eye Hospital of Wenzhou Medical University (No.2019-077-K-76-01). All subjects were recruited and completed corresponding follow-ups between 2020 and 2023. All subjects and their guardians signed the written consent forms prior to the start of the study and had the right to terminate this study at any time.

### Subjects

Children, 8–14 years of age, with myopia (spherical component) between 0.75 dioptre (D) and 5.00 D (inclusive), cylinder of ≤ 1.50 D, anisometropia of ≤ 1.00 D, best-corrected visual acuity of 0.0 logarithm of the minimum angle of resolution or better in both eyes were recruited. Those who had used other myopia control methods or had any contraindications to overnight orthokeratology were excluded.

### OKL fitting and procedures

The lenses were Dreamlite VST designs (Procornea Nederland B.V.) and were manufactured with Boston XO whose oxygen permeability (DK) was $100\times 10^{-11}\left(\frac{cm^{2}}{s}\right)\left[\frac{mLO_{2}}{\left(mL\times mmHg\right)}\right]$.

The parameters of ordered OKL were determined according to the diagnostic lenses that provided the best fit. Follow-ups were scheduled at 1 day, 1 week, 1 month, and every 3 months after orthokeratology. The follow-up examinations included uncorrected visual acuity, slit lamp examination, fitting assessment, intraocular pressure and corneal topography. AL was measured with LenStar LS900 (Haag-Streit, Koeniz, Switzerland) at six-month intervals. All AL measurements were performed between 8:00 and 10:00 a.m. to minimize the influence of diurnal variation (20). Subjects with serious lens decentration or complications were discontinued from the study.

### Peripheral refraction measurement

The peripheral refraction was measured using an infrared autorefractor (Grand Seiko WAM-5500, Hiroshima, Japan). The auxiliary device consisted of two circular arcs with a radius of curvature of 33 cm. The Maltese cross targets were positioned at an angle of 0° (central), 10°, 20°, 30° from the visual axis in the horizontal visual field and 5°, 10°, 15° from the visual axis in the vertical visual field (21). The horizontal measurement range was 30° from the visual axis, while the vertical range was 15° from the visual axis due to constraints in eye movement and orbital anatomy.

The measurement steps were as follows: first, subjects were administered with Tropicamide eye drops for mydriasis. Their heads were firmly placed against the forehead rest, and their chins were placed in the chin rest. Then the subjects were instructed to keep their head still and rotate their eyes to gaze at different targets in horizontal or vertical meridian. To ensure the accuracy of measurements, the axis of the autorefractor was aligned with the geographic center of the pupil and the fellow eye was occluded. Each location was measured at least 5 times.

Refractive parameters (nasal 10 °, 20 °, 30 °, temporal 10 °, 20 °, 30 °, superior 5 °, 10 °, 15 °, inferior 5 °, 10 °, 15 °, and on-axis) were measured at baseline and every 6 months after orthokeratology. These refractive parameters included spherical refractive error (Sph), cylinder (Cyl) and its axis (θ), which were converted into on-axis spherical equivalent refraction (on-axis SER), peripheral refraction (PR), RPR, vertical (90°)/horizontal (180°) astigmatism component (J_0_), and oblique (45° and 135°) astigmatism component (J_45_). The calculation formulas were as follows (22):


$$\begin{array}{l} PR=Sph+\frac{Cyl}{2} \end{array}$$

$$\begin{array}{l} RPR=PR-on-axis\;SER \end{array}$$

$$\begin{array}{l} J_{0}=\frac{-Cyl}{2}\times \cos2\theta \end{array}$$

$$\begin{array}{l} J_{45}=\frac{-Cyl}{2}\times \sin2\theta \end{array}$$

### Statistical analysis

Only the right eye data were included in analyses. Statistical analysis was performed with SPSS software, version 26 (IBM Corp., Armonk, NY, USA). Descriptive statistics were calculated for age, gender, AL, and refractive parameters. Normality was assessed using the Shapiro-Wilk test. If the data followed a normal distribution, then parametric tests were used; otherwise non-parametric tests were employed. For the significance test of changes in AE and peripheral refraction, one-way repeated measures analysis of variance (ANOVA) was used. Bonferroni pairwise multiple comparison *t*-test was used for *post-hoc* pairwise comparison. Pearson's correlation test was used to assess the correlation between AE and RPR, J_0_, J_45_. Multiple linear regression analysis was used to analyze the effects of peripheral refraction on AE.

## Results

A total of 39 subjects were recruited in this study, including 23 males and 16 females, with mean age of 10.64 ± 1.98 years and spherical equivalent refraction of −2.77 ± 1.07 D. 35, 32, 24, and 29 subjects completed 6-, 12-, 18- and 24-month follow-up, respectively. The main reason for the losses to follow-up was the strict management and control of the COVID-19 pandemic in China.

### Changes in AL and peripheral refraction

**Figure 1:**
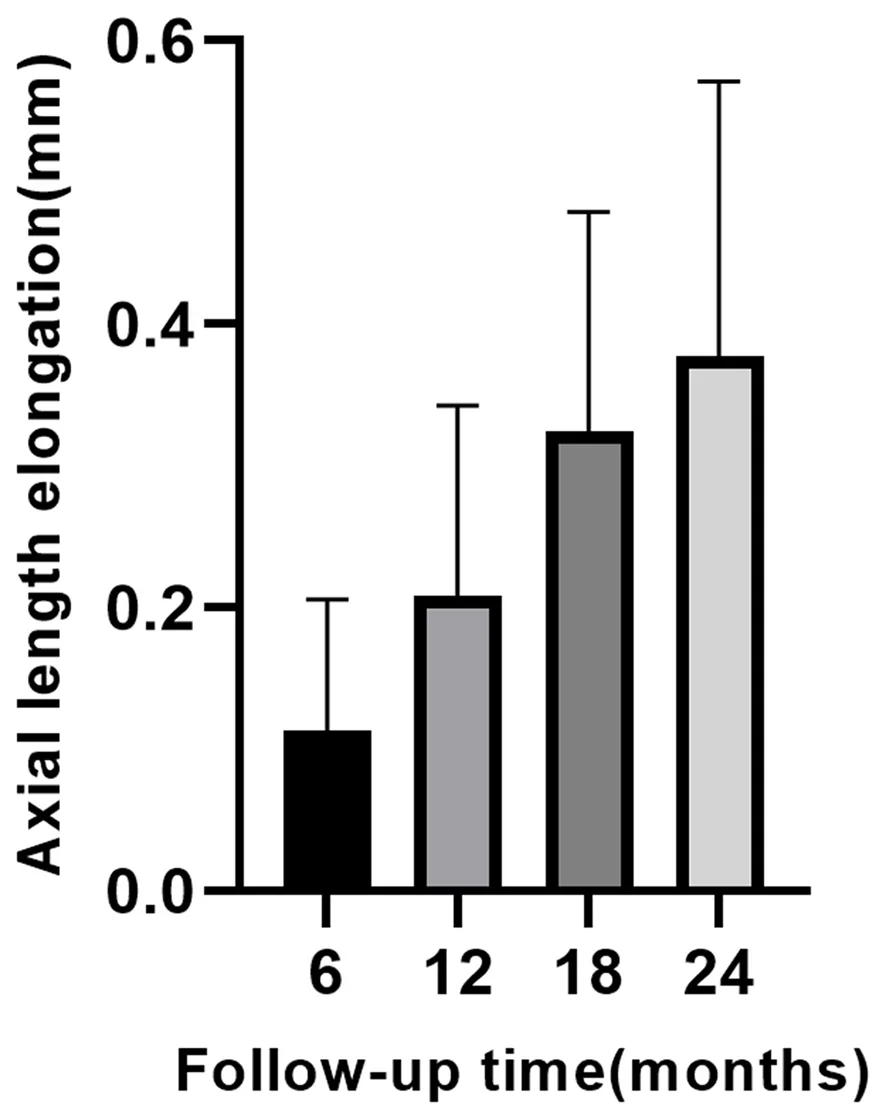
Axial elongation of every 6-month follow-up.

The mean AL was 25.01 ± 0.84 mm at baseline and was 25.41 ± 0.78 mm at 24-month follow-up. The AE was 0.11 ± 0.09 mm, 0.21 ± 0.13 mm, 0.32 ± 0.14 mm and 0.38 ± 0.17 mm at each follow-up, respectively (Figure 1). The AL increased significantly after orthokeratology (*p* < 0.001, one-way repeated measures ANOVA), and there were also differences in AE between every 6-month follow-up (all *p* ≤ 0.017, Bonferroni *t*-test).

**Figure 2:**
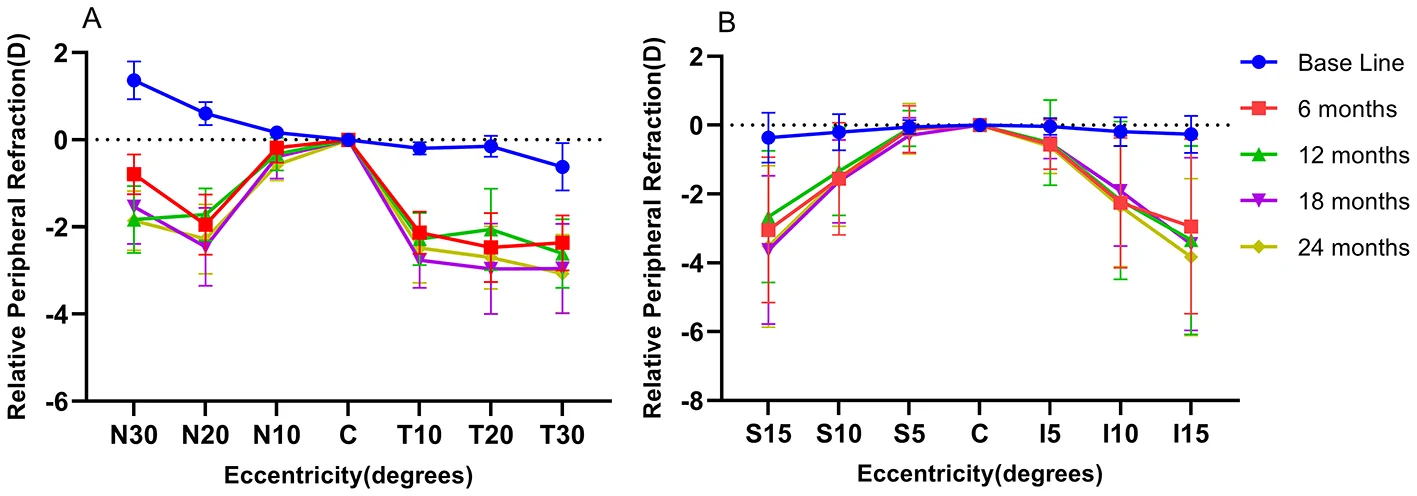
Mean relative peripheral refraction along the **(A)** horizontal and **(B)** vertical meridians at baseline and after orthokeratology. Error bars represent the standard error of the mean. T, temporal; N, nasal; S, superior; I, inferior; D, dioptre.

**Figure 3:**
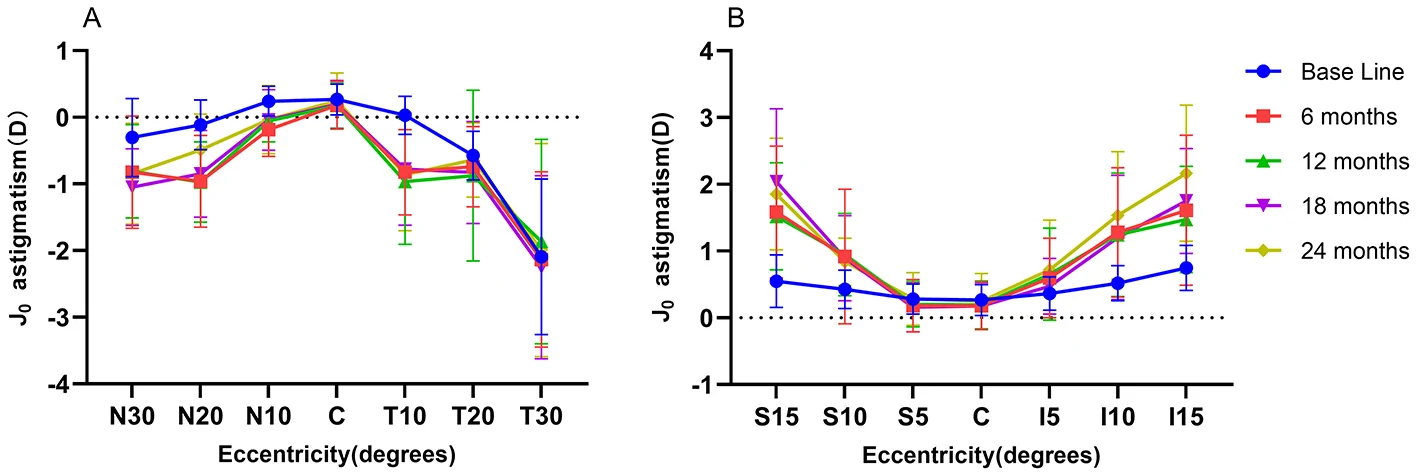
Mean J_0_ astigmatism along the **(A)** horizontal and **(B)** vertical meridians at baseline and after orthokeratology. Error bars represent the standard error of the mean. T, temporal; N, nasal; S, superior; I, inferior; D, dioptre.

**Figure 4:**
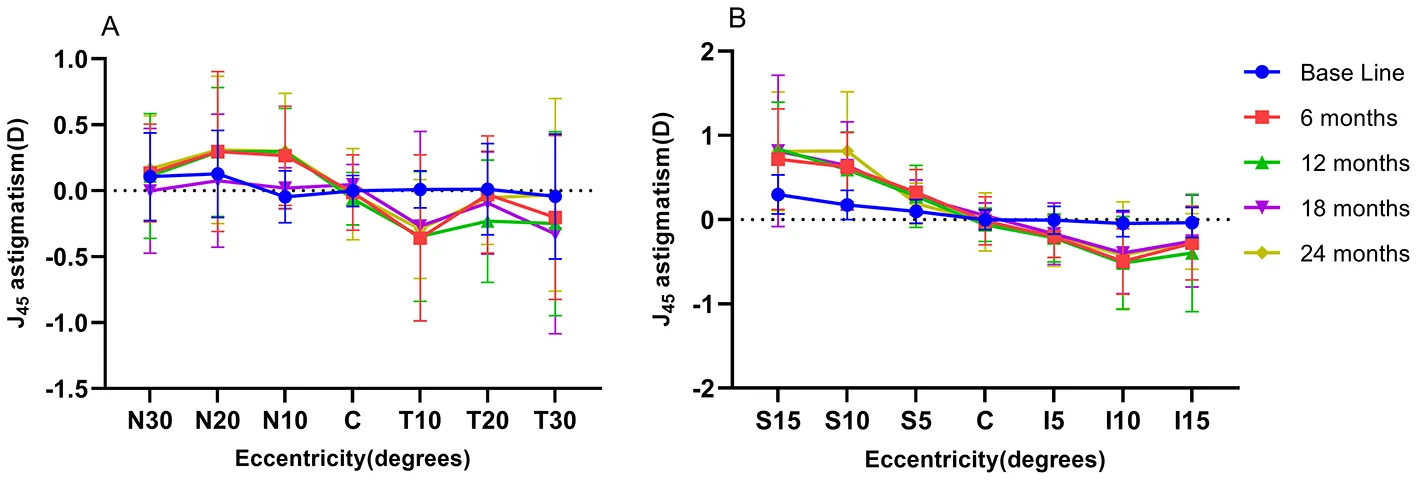
Mean J_45_ astigmatism along the **(A)** horizontal and **(B)** vertical meridians at baseline and after orthokeratology. Error bars represent the standard error of the mean. T, temporal; N, nasal; S, superior; I, inferior; D, dioptre.

The baseline data and changes in central and peripheral retinal refractive parameters after orthokeratology are shown in Table 1. Except the superior 5° (S5°), RPR showed significant myopic defocus at all locations (all *p* ≤ 0.003, one-way repeated measures ANOVA) (Figure 2), with no further significant change between 6- and 24-month follow-up except N30° at 6 months (all *p* ≥ 0.055, Bonferroni *t*-test). Except T20° and T30°, J_0_ changed significantly after orthokeratology (all *p* ≤ 0.045, one-way repeated measures ANOVA) (Figure 3), and there was no further significant change (all *p* ≥ 0.094, Bonferroni *t*-test) except N20°, S15° and inferior 15° (I15°). Except N30°, J_45_ changed significantly (all *p* ≤ 0.022, one-way repeated measures ANOVA) (Figure 4) after orthokeratology, and without any further significant change (all *p* ≥ 0.059, Bonferroni t-test) throughout the study period except T20° and N10°.

**Table 1:** Baseline data and changes in central and peripheral retinal refractive parameters after orthokeratology.

Parameter	Mean ±SD(D)				
	Base line	6 months	12 months	18 months	24 months
On-axis SER	−3.05 ± 1.17	−1.47 ± 1.15	−1.50 ± 1.09	−1.31 ± 1.17	−1.51 ± 0.79
On-axis J_0_	0.27 ± 0.23	0.18 ± 0.36	0.19 ± 0.36	0.18 ± 0.35	0.25 ± 0.42
On-axis J_45_	−0.00 ± 0.12	−0.01 ± 0.29	−0.06 ± 0.20	−0.05 ± 0.15	−0.03 ± 0.35
T10° RPR	−0.20 ± 0.43	−2.13 ± 1.39	−2.28 ± 1.66	−2.77 ± 1.46	−2.48 ± 2.06
T10° J_0_	0.03 ± 0.29	−0.82 ± 0.64	−0.78 ± 0.84	−0.97 ± 0.94	−0.86 ± 0.84
T10° J_45_	0.01 ± 0.14	−0.36 ± 0.64	−0.35 ± 0.49	−0.27 ± 0.72	−0.29 ± 0.37
T20° RPR	−0.15 ± 0.73	−2.47 ± 2.27	−2.06 ± 2.59	−2.96 ± 2.45	−2.70 ± 1.86
T20° J_0_	−0.57 ± 0.36	−0.74 ± 0.60	−0.83 ± 0.77	−0.88 ± 1.28	−0.64 ± 0.56
T20° J_45_	0.01 ± 0.35	−0.03 ± 0.44	−0.23 ± 0.46	−0.09 ± 0.39	−0.05 ± 0.36
T30° RPR	−0.62 ± 1.68	−2.36 ± 1.84	−2.61 ± 2.18	−2.96 ± 2.43	−3.07 ± 2.31
T30° J_0_	−2.10 ± 1.17	−2.13 ± 1.32	−2.25 ± 1.37	−1.87 ± 1.53	−1.99 ± 1.60
T30° J_45_	−0.04 ± 0.47	−0.20 ± 0.62	−0.25 ± 0.70	−0.33 ± 0.76	−0.03 ± 0.73
N10° RPR	0.16 ± 0.41	−0.19 ± 0.92	−0.33 ± 1.03	−0.39 ± 1.13	−0.58 ± 0.92
N10° J_0_	0.24 ± 0.22	−0.19 ± 0.40	−0.04 ± 0.45	−0.06 ± 0.31	−0.04 ± 0.51
N10° J_45_	−0.05 ± 0.20	0.26 ± 0.38	0.30 ± 0.33	0.02 ± 0.15	0.30 ± 0.44
N20° RPR	0.60 ± 0.80	−1.95 ± 2.00	−1.72 ± 1.61	−2.46 ± 2.12	−2.28 ± 2.06
N20° J_0_	−0.11 ± 0.37	−0.96 ± 0.69	−0.85 ± 0.65	−0.97 ± 0.60	−0.49 ± 0.54
N20° J_45_	0.13 ± 0.33	0.30 ± 0.61	0.29 ± 0.49	0.08 ± 0.50	0.31 ± 0.56
N30° RPR	1.36 ± 1.34	−0.79 ± 1.25	−1.83 ± 2.13	−1.54 ± 2.02	−1.86 ± 1.75
N30° J_0_	−0.30 ± 0.59	−0.82 ± 0.84	−1.05 ± 0.58	−0.81 ± 0.70	−0.85 ± 0.76
N30° J_45_	0.11 ± 0.33	0.13 ± 0.37	0.11 ± 0.47	−0.00 ± 0.47	0.17 ± 0.40
S5° RPR	−0.06 ± 0.20	−0.12 ± 0.68	−0.09 ± 0.51	−0.29 ± 0.51	−0.11 ± 0.73
S5° J_0_	0.28 ± 0.23	0.18 ± 0.39	0.20 ± 0.34	0.16 ± 0.37	0.28 ± 0.39
S5° J_45_	0.10 ± 0.14	0.33 ± 0.27	0.28 ± 0.37	0.27 ± 0.20	0.19 ± 0.24
S10° RPR	−0.20 ± 0.52	−1.56 ± 1.63	−1.34 ± 1.28	−1.63 ± 1.20	−1.52 ± 1.42
S10° J_0_	0.43 ± 0.29	0.92 ± 1.01	0.95 ± 0.62	0.89 ± 0.64	0.84 ± 0.35
S10° J_45_	0.17 ± 0.17	0.62 ± 0.41	0.59 ± 0.45	0.64 ± 0.52	0.82 ± 0.70
S15° RPR	−0.36 ± 0.72	−3.04 ± 2.11	−2.66 ± 1.91	−3.63 ± 2.16	−3.52 ± 2.35
S15° J_0_	0.55 ± 0.39	1.58 ± 0.99	1.52 ± 0.80	2.04 ± 1.09	1.85 ± 0.84
S15° J_45_	0.30 ± 0.23	0.72 ± 0.60	0.84 ± 0.56	0.82 ± 0.90	0.81 ± 0.70
I5° RPR	−0.04 ± 0.24	−0.56 ± 0.71	−0.51 ± 1.24	−0.53 ± 0.44	−0.62 ± 0.78
I5° J_0_	0.36 ± 0.25	0.60 ± 0.59	0.65 ± 0.69	0.47 ± 0.42	0.72 ± 0.75
I5° J_45_	−0.01 ± 0.16	−0.20 ± 0.25	−0.22 ± 0.29	−0.17 ± 0.36	−0.20 ± 0.36
I10° RPR	−0.19 ± 0.42	−2.26 ± 1.89	−2.19 ± 2.29	−1.91 ± 1.60	−2.36 ± 1.75
I10° J_0_	0.52 ± 0.26	1.28 ± 0.97	1.24 ± 0.92	1.20 ± 0.93	1.54 ± 0.96
I10° J_45_	−0.05 ± 0.15	−0.50 ± 0.39	−0.51 ± 0.55	−0.40 ± 0.48	−0.42 ± 0.63
I15° RPR	−0.27 ± 0.54	−2.94 ± 2.54	−3.34 ± 2.74	−3.45 ± 2.51	−3.83 ± 2.28
I15° J_0_	0.75 ± 0.34	1.61 ± 1.12	1.47 ± 0.80	1.75 ± 0.79	2.17 ± 1.02
I15° J_45_	−0.03 ± 0.18	−0.28 ± 0.44	−0.40 ± 0.70	−0.25 ± 0.54	−0.26 ± 0.33

RPR, relative peripheral refraction; T, temporal; N, nasal; S, superior; I, inferior; D, dioptre; SER, spherical equivalent refraction; J_0_, vertical (90°)/horizontal (180°) astigmatism component; J_45_, oblique (45° and 135°) astigmatism component; SD, standard deviation.

### Relationship between AE and peripheral refraction

**Figure 5:**
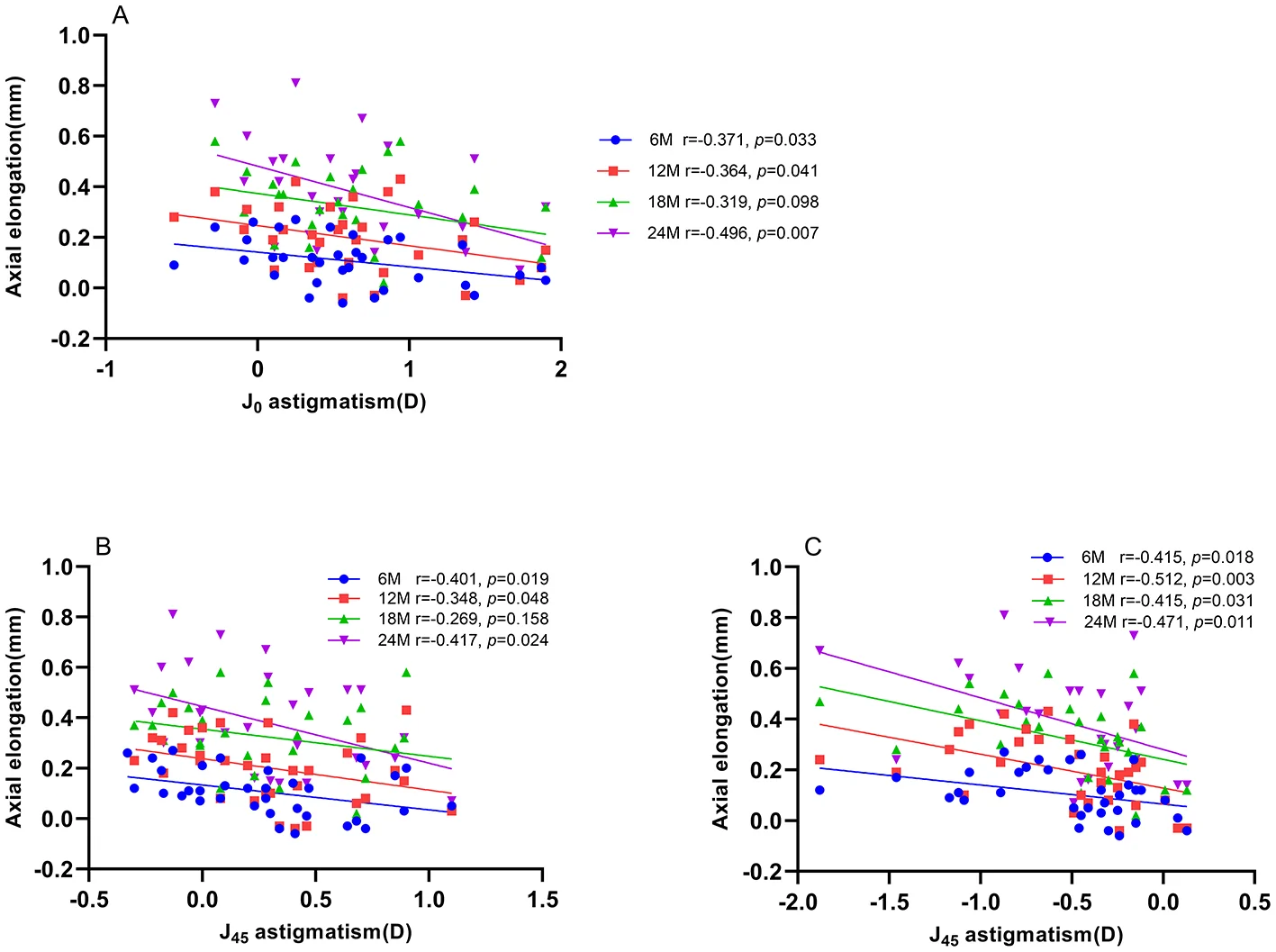
**(A)** Correlation between axial elongation of each follow-up and the 6-month J_0_ astigmatism at inferior 5° after orthokeratology; **(B)** Correlation between axial elongation of each follow-up and the 6-month J_45_ astigmatism at nasal 10° after orthokeratology; **(C)** Correlation between axial elongation of each follow-up and the 6-month J_45_ astigmatism at inferior 10° after orthokeratology. M, month. D, dioptre.

Tables 2–4 shows the correlations between AE of each follow-up and the 6-month RPR, J_0_ and J_45_ after orthokeratology, respectively. Among them, the 6-month J_0_ at I5°, J_45_ at N10°, J_45_ at I10° had the relatively consistent correlations with AE of each follow-up (Figure 5). So we used the three peripheral refractive parameters to build predictive models for AE after orthokeratology with multiple linear regression analysis (Table 5). Except 18 months (*r*^2^ = 0.263, *p* = 0.077), 6, 12, 24 months' predictive models were constructed (*r*^2^ = 0.340, 0.336, 0.430, *p* = 0.010, 0.013, 0.004, respectively) and the 6-month J_45_ at I10° had the greatest impact on the AEs (standardized beta = −0.404, −0.445, −0.412; *p* = 0.018, 0.012, 0.016, respectively).

**Table 2:** Correlation analyses between AE of each follow-up and the 6-month relative peripheral refraction.

Parameter	Item	T10°	T20°	T30°	N10°	N20°	N30°	S5°	S10°	S15°	I5°	I10°	I15°
AE 6months	r	−0.027	0.045	0.087	0.121	0.052	0.026	−0.384	−0.217	−0.118	0.151	0.038	−0.013
	*p*	0.880	0.802	0.623	0.525	0.769	0.892	**0.027** ^*^	0.218	0.505	0.418	0.831	0.941
AE 12 months	r	−0.026	0.058	−0.157	0.076	0.026	0.003	−0.298	−0.254	−0.131	0.066	−0.028	0.028
	*p*	0.886	0.752	0.382	0.694	0.886	0.990	0.098	0.153	0.467	0.730	0.879	0.877
AE 18 months	r	0.040	−0.008	−0.254	0.085	−0.062	−0.025	−0.307	−0.277	−0.217	−0.056	−0.230	−0.114
	*p*	0.839	0.966	0.183	0.681	0.751	0.904	0.112	0.146	0.257	0.787	0.231	0.557
AE 24 months	r	0.210	0.014	−0.084	0.133	0.106	0.137	−0.305	−0.389	−0.264	0.259	−0.092	−0.044
	*p*	0.283	0.942	0.665	0.510	0.584	0.496	0.115	**0.037** ^*^	0.166	0.202	0.635	0.823

T, temporal; N, nasal; S, superior; I, inferior; AE, axial elongation. ^*^Significant effect on axial elongation. Values in bold represent *P*-values less than 0.05.

**Table 3:** Correlation analyses between AE of each follow-up and the 6-month J_0_ astigmatism.

Parameter	Item	T10°	T20°	T30°	N10°	N20°	N30°	S5°	S10°	S15°	I5°	I10°	I15°
AE 6months	r	−0.220	0.049	−0.193	−0.026	−0.066	−0.042	0.038	0.046	0.089	−0.371	−0.186	−0.002
	*p*	0.219	0.797	0.274	0.890	0.715	0.813	0.833	0.797	0.617	**0.033** ^*^	0.292	0.992
AE 12 months	r	−0.206	0.217	−0.367	−0.184	−0.158	−0.106	0.004	−0.075	−0.103	−0.364	−0.234	−0.12
	*p*	0.257	0.259	**0.036** ^*^	0.331	0.387	0.557	0.985	0.679	0.570	**0.041** ^*^	0.190	0.506
AE 18 months	r	−0.109	0.159	−0.246	−0.22	−0.002	−0.387	−0.041	−0.064	0.004	−0.319	−0.026	−0.071
	*p*	0.581	0.447	0.197	0.270	0.990	**0.038** ^*^	0.835	0.740	0.983	0.098	0.893	0.716
AE 24 months	r	−0.082	0.066	−0.197	−0.11	−0.189	−0.302	−0.016	0.065	0.024	−0.496	−0.149	0.013
	*p*	0.678	0.750	0.306	0.585	0.337	0.111	0.936	0.738	0.902	**0.007** ^*^	0.439	0.948

T, temporal; N, nasal; S, superior; I, inferior; AE, axial elongation. ^*^Significant effect on axial elongation. Values in bold represent *P*-values less than 0.05.

**Table 4:** Correlation analyses between AE of each follow-up and the 6-month J_45_ astigmatism.

Parameter	Item	T10°	T20°	T30°	N10°	N20°	N30°	S5°	S10°	S15°	I5°	I10°	I15°
AE 6months	r	0.220	0.086	−0.250	−0.401	−0.210	−0.239	0.098	0.272	0.106	−0.058	−0.415	−0.291
	*p*	0.212	0.629	0.154	**0.019** ^*^	0.234	0.174	0.581	0.133	0.558	0.750	**0.018** ^*^	0.101
AE 12 months	r	0.184	0.033	−0.234	−0.348	−0.117	−0.119	0.165	0.022	0.030	−0.088	−0.512	−0.309
	*p*	0.306	0.857	0.190	**0.048** ^*^	0.517	0.509	0.358	0.910	0.877	0.663	**0.003** ^*^	0.086
AE 18 months	r	0.036	−0.134	−0.103	−0.269	0.124	−0.102	0.014	−0.18	−0.07	0.116	−0.415	−0.154
	*p*	0.854	0.487	0.594	0.158	0.522	0.599	0.944	0.400	0.736	0.579	**0.031** ^*^	0.434
AE 24 months	r	0.261	0.029	−0.229	−0.417	−0.028	0.067	0.139	0.03	−0.015	0.074	−0.471	−0.25
	*p*	0.172	0.879	0.233	**0.024** ^*^	0.884	0.728	0.471	0.890	0.941	0.727	**0.011** ^*^	0.200

T, temporal; N, nasal; S, superior; I, inferior; AE, axial elongation. ^*^Significant effect on axial elongation. Values in bold represent *P*-values less than 0.05.

**Table 5:** Multivariate linear regression analyses of AE of each follow-up and the 6-month J_0_ at I5°, J_45_ at N10°, J_45_ at I10°.

Parameter	AE 6 months			AE 12 months			AE 18 months			AE 24 months		
	standardized beta	Beta	*p*	standardized beta	Beta	*P*	standardized beta	Beta	*p*	standardized beta	Beta	*p*
I5° J_0_	−0.074	0.012	0.727	−0.145	−0.032	0.499	−0.240	−0.062	0.314	−0.359	−0.118	0.087
N10° J_45_	−0.341	0.085	0.110	−0.211	−0.076	0.318	−0.127	−0.050	0.591	−0.178	−0.095	0.385
I10° J_45_	−0.404	0.102	**0.018**	−0.445	−0.154	**0.012**	−0.392	−0.173	**0.045**	−0.412	−0.180	**0.016**
r^2^(*p*)	0.340(**0.010**)			0.336(**0.013**)			0.263(0.077)			0.430(**0.004**)		

N, nasal; I, inferior; AE, axial elongation; J_0_, vertical (90°)/horizontal (180°) astigmatism component; J_45_, oblique (45° and 135°) astigmatism component. Values in bold represent *P*-values less than 0.05.

## Discussion

### Changes in relative peripheral refraction

This study found that horizontal RPR showed significant myopic defocus after orthokeratology, consistent with previous studies (15, 23, 24). Moreover, except N30° of 6 months, there was no further significant change in the horizontal RPR, which was consistent with the studies by Wang et al. (17) and Jackson et al. (14). However, Wang did not measure the RPR at 6 and 18 months, while Jackson did not measure the RPR at 24 months. Therefore, our study further confirmed that the changes in horizontal RPR caused by OKL remained stable during a long-term (at least 1 year) orthokeratology.

In the vertical meridian, except S5°, RPR of all locations showed myopic defocus at 6 months after orthokeratology. There have been a few studies on the vertical RPR after orthokeratology. Kang and Swarbrick (13) found that the vertical RPR experienced a myopic defocus except at S10°. Ticak's study (18) also showed that there was significant myopic defocus observed vertically except at I10°, whereas Queirós et al. (19) reported significant myopic defocus only at S15°. These discrepancies may be attributed to differences in OKL wear duration and study populations, as the aforementioned studies primarily examined adults after short-term orthokeratology (no more than 1 month). Moreover, our study found that vertical RPR remained stable between the 6- and 24-month follow-ups, consistent with the horizontal meridian results. This indicates that the myopic defocus induced by OKL across different meridians remains stable and appears unaffected by axial elongation.

### Changes in peripheral astigmatism components

Except T20° and T30°, significant negative shift in J_0_ was observed in the horizontal meridian after orthokeratology, which was basically consistent with previous studies (13, 19, 23) except a few locations. In addition, this study found that J_45_ of all horizontal locations changed significantly after orthokeratology, with negative shift temporally and positive shift nasally, which was consistent with Kang's findings (13) but differed from those of Charman et al. (23) and Queirós et al. (19). These discrepancies may be attributed to differences in the study populations and wear duration, given that prior researches focused on adults in short-term orthokeratology, whereas this study involved children over a longer period.

In the vertical meridian, we also found significant changes in J_0_ and J_45_ at all locations after orthokeratology. When J_0_ showed positive shift on both sides, J_45_ showed inconsistent trends, with positive shift superiorly and negative shift inferiorly. Interestingly, unlike RPR only showing myopic defocus, which means more negative in peripheral refraction, J_0_ and J_45_ exhibited positive shift in both horizontal and vertical meridians. This appears to contradict the conventional notion that myopic defocus inhibits myopia progression. However, a recent study (25) found that both peripheral myopic defocus lenses and hyperopic defocus lenses could slow myopia progression, with no significant difference in efficacy. We therefore speculate that a similar principle may apply to the astigmatism component of peripheral refraction, or that its mechanism in myopia control differs from that of RPR.

Unlike the RPR, which remained stable, J_0_ and J_45_ showed alterations at some locations during the follow-up period after orthokeratology. Previous studies have shown that the myopia control efficacy of optical interventions was greater in the first year and decreased over time (26), possibly due to the neural adaptability of the retina. The prolonged use of the same optical intervention may trigger an adaptive neural response, leading to a decrease in retinal sensitivity to the myopia slowing signals (27). However, this attenuation of efficacy was not observed in the present study. We speculate that this is due to the alterations of certain peripheral astigmatism components after orthokeratology, which leads to the refreshing of defocus signals on the retina, thereby preventing neural adaptation and contributing to persistent myopia control.

### Peripheral refraction as predictors of myopia progression

Another interesting finding was that the AE of each follow-up was negatively correlated with J_0_ at I5°, J_45_ at N10° and J_45_ at I10° measured at 6 months after orthokeratology. Among them, the correlations between 18-month AE and J_0_ at I5° as well as J_45_ at N10° were not statistically significant, but their *p*-values approached 0.05. This may be due to the highest number of missed visits at 18 months because of the COVID-19 pandemic and stringent control measures in China. So more samples are required in future studies to verify these correlations.

Simultaneously, we attempted to establish a regression model between the AE of each follow-up and the three 6-month peripheral refraction. Except the 18-month follow-up, predictive models were successfully established for all other follow-ups. Relatively few studies have attempted to use early peripheral refraction to predict long-term AE after orthokeratology. Jakobsen et al. (14) found that the AE of 18 months was correlated with baseline horizontal PRs and the RPR of N30°. But they didn't find any correlation between AE and the changes in PR or RPR at 6 months during 18-month follow-up at any eccentricity. Chen et al. (16) found that the AE was correlated with the RPRs of T30° and N30° at 3 months after orthokeratology. However, they did not further explain whether these correlations could be used to predict long-term AE, even though their total follow-up period was 12 months.

Moreover, previous studies have rarely conducted correlation analysis specifically on the astigmatism component of peripheral refraction. Our study found that, compared to RPR, the astigmatism component might play a more significant role in controlling myopia progression. This is supported by Hosein et al. (28), who found that even short-term (1 h) wear of astigmatism lenses significantly alters choroidal thickness, an important factor known to influence ocular growth. Smith's study in monkeys (29) showed that in mixed astigmatism (tangential foci located anterior and sagittal foci located posterior to retina), ocular growth occurred to reposition the retina toward the posteriorly located focus. But in compound astigmatism, in which the tangential and sagittal foci were located posterior to the retina, the ocular growth acted to reposition the retina toward the less posterior foci. A potential explanation is that ganglion cells in the retina are sensitive to orientation and could recognize different astigmatism signals to regulate ocular growth (30, 31). Therefore we cannot ignore the role of peripheral astigmatism components in controlling myopia progression.

In addition, correlation analysis revealed that only a few peripheral retinal locations exhibited relatively consistent associations between their astigmatism components and axial elongation (AE). Moreover, different astigmatism components contributed variably to AE, with the J_45_ at I10° showing the strongest contribution in multiple linear regression models—a finding that remained consistent in the 18-month model. This suggests that astigmatism components in different peripheral retinal regions may have distinct effects on AE. Swiatczak et al. (32) found that the 6°-10° peripheral retina was most sensitive to defocus signals and could regulate ocular growth independently. There could be some specific regions in the peripheral retina that are particularly sensitive or responsive to the orientation and magnitude of astigmatism signals (33). These insights could inform the future development of personalized orthokeratology lenses or peripheral defocus spectacle designs.

One limitation of this study was the relatively small sample size and losses of visits at each follow-up due to the COVID-19 pandemic. Therefore, future studies with larger samples are needed. Another limitation was that we did not measure the earlier peripheral refraction after orthokeratology, such as 3 months or 1 month. Further studies are needed to investigate whether we can use the earlier peripheral refraction to predict the myopia control efficacy of OKL, so as to evaluate the long-term control efficacy and intervene as early as possible.

## Conclusion

In summary, our study found that orthokeratology could significantly change the peripheral refraction in both horizontal and vertical meridians, including RPR and astigmatism components. Through correlation analysis, the peripheral astigmatism components at certain locations of 6 months could serve as predictive indicators for long-term myopia control efficacy of orthokeratology. In the future, we need to pay more attention to the impact of peripheral astigmatism components on myopia control.

## Data Availability

The original contributions presented in the study are included in the article/supplementary material, further inquiries can be directed to the corresponding author.
